# Supplementary material for: CircAFF1 Is a Circular RNA with a Role in Alveolar Rhabdomyosarcoma Cell Migration
Source: Biomedicines. 2023 Jul 4;11(7):1893. doi: 10.3390/biomedicines11071893 (PMC10376778; doi:10.3390/biomedicines11071893)
Supplement: Supplementary file 1 [file biomedicines-11-01893-s001.zip › Supplementary figures.pdf]

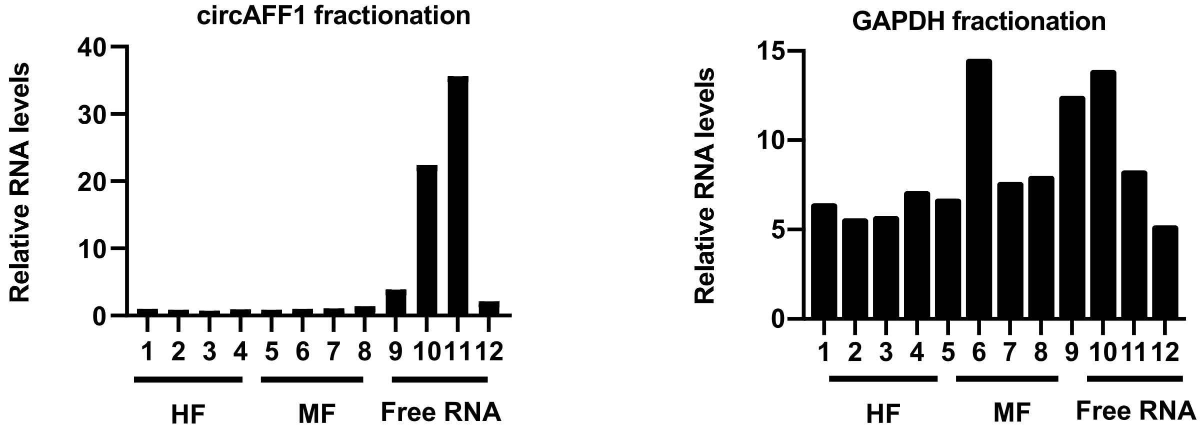

Figure S1 Relative levels of RNA detected by qRT-PCR of circAFF1 and GAPDH mRNA in RH4 After sucrose polysome fractionation.

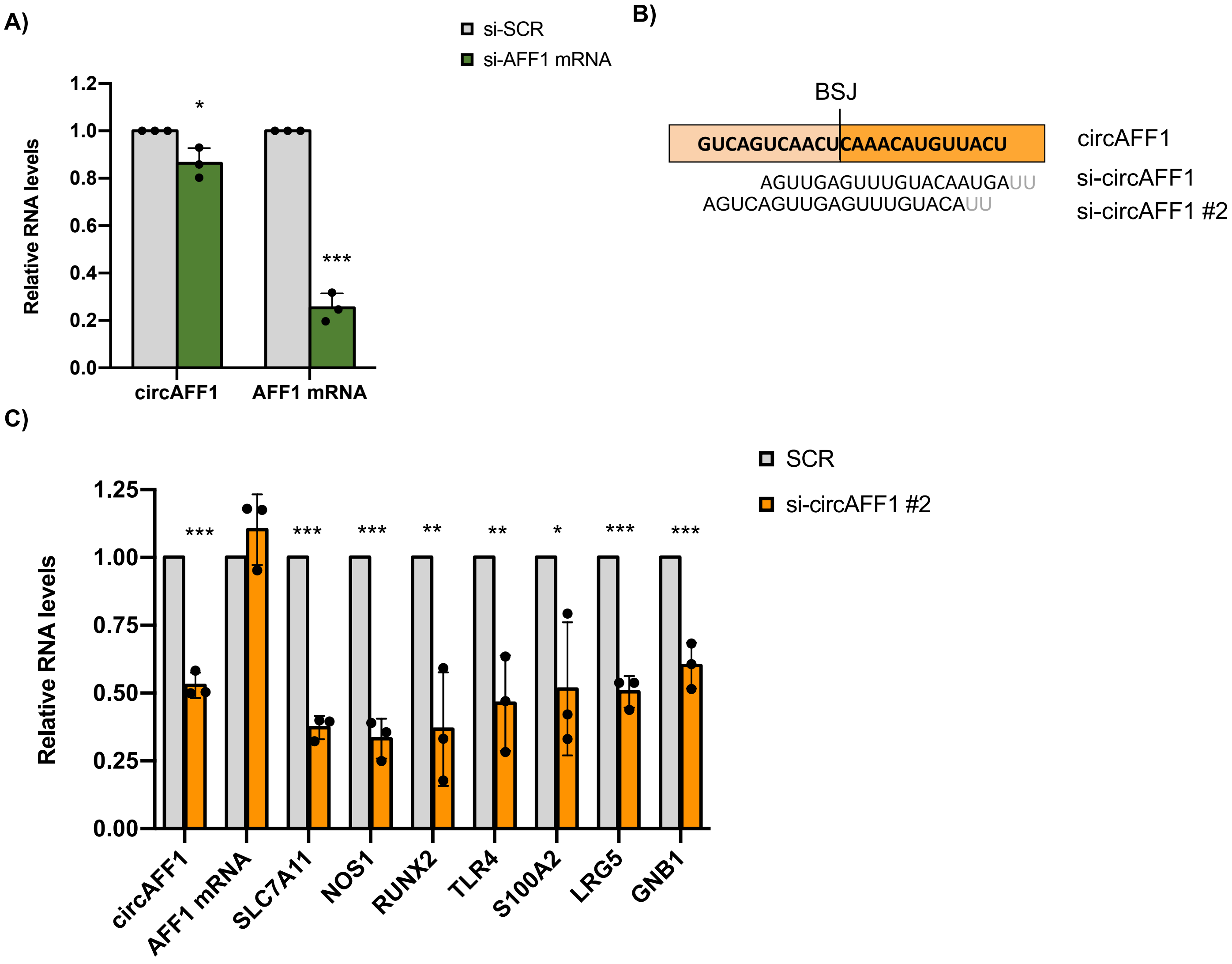

Figure S2 (**A**) Relative levels of RNA normalised against GAPDH transcript detected by qRT-PCR of circAFF1 and AFF1 mRNA in RH4 cells treated with si-SCR or si-AFF1 mRNA. Data are represented as the mean of fold changes ± standard deviation of at least 3 biological replicates. (**B**) Cartoon depiction the sequence the design strategy for si-circAFF1 #2 (**C**) Relative levels of RNA normalised against GAPDH transcript detected by qRT-PCR of circAFF1 and AFF1 mRNA in RH4 cells treated with si-SCR or si-circAFF1 #2. Data are represented as the mean of fold changes ± standard deviation of at least 3 biological replicates.

Individual datapoints represented as black dots. Where statistical analysis was performed, the ratio of each sample vs. its experimental control was tested by a two-tailed unpaired Student's t-test. *: p-value < 0.05, **: p-value < 0.01, ***: p-value < 0.001.

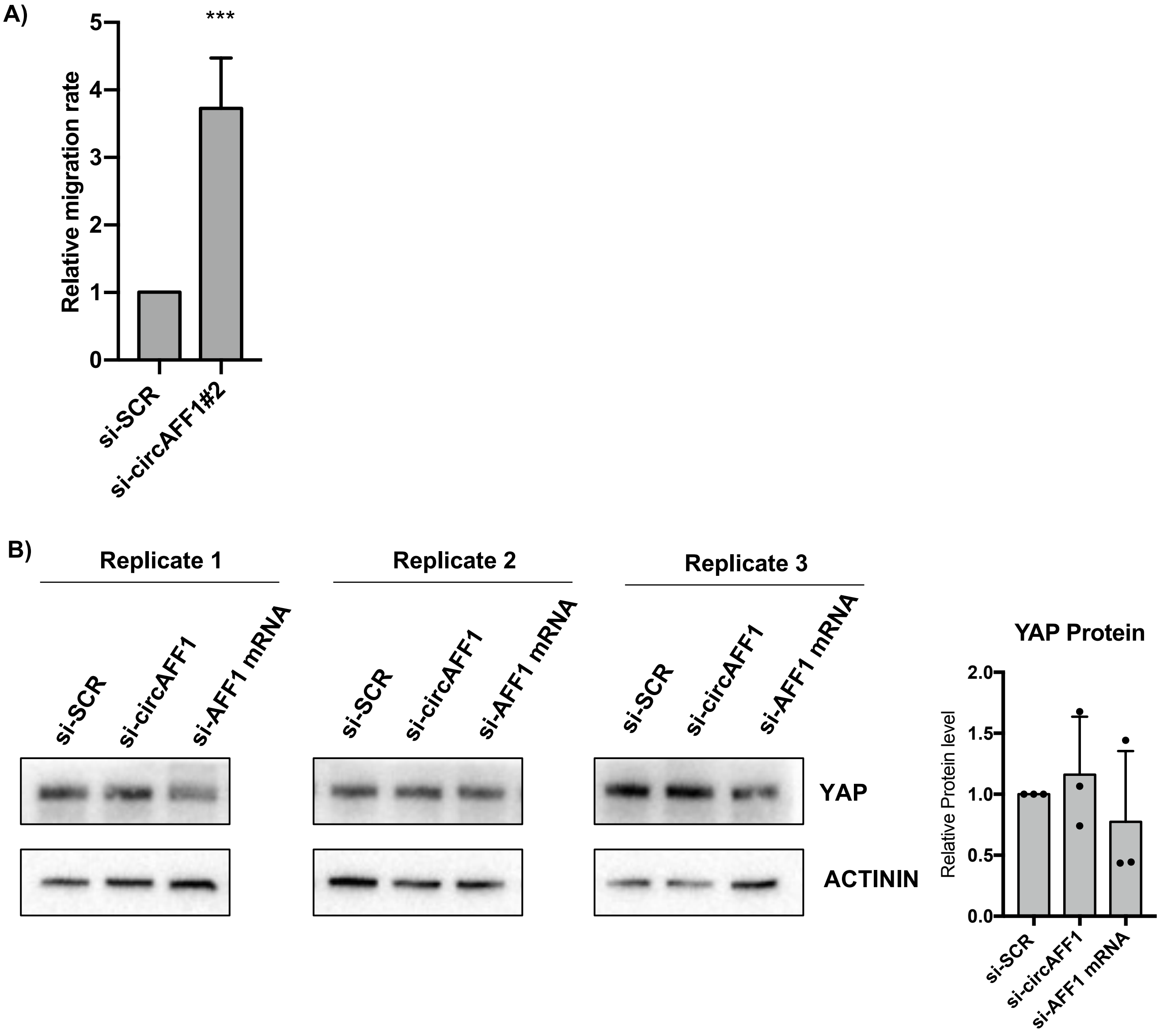

Figure S3 (**A**) Right panel: Relative migration rate of RH4 cells upon si-SCR or si-circAFF1 #2. Data are depicted as the average of fold changes ± SD of 3 biological replicates. **(B)** Protein levels of YAP1 and Actinin in RH4 cells after si-SCR , si-circAFF1 or ci-AFF1 mRNA treatment. Right panel: Relative to actinin quantification of three independent experiments. Data are represented as the mean of fold changes ± standard deviation of biological replicates. Individual datapoints represented as black dots. Where statistical analysis was performed, the ratio of each sample vs. its experimental control was tested by a two-tailed unpaired Student’s t-test. ***: p-value < 0.001.
